# Clinical Value of Anti-Integrin αvβ6 Antibody Serum-Level Measurement in Inflammatory Bowel Diseases

**DOI:** 10.3390/jcm15030948

**Published:** 2026-01-24

**Authors:** Dorottya Angyal, Fruzsina Balogh, Lorant Gonczi, Livia Lontai, Janos P. Kosa, Nora Garam, Peter L. Lakatos, Akos Ilias

**Affiliations:** 1 Department of Internal Medicine and Oncology, Semmelweis University, 1083 Budapest, Hungary; 2 Division of Gastroenterology, Central Hospital of Northern Pest, Military Hospital, 1134 Budapest, Hungary; 3 Division of Gastroenterology, McGill University, Montreal, QC H4A 3J1, Canada

**Keywords:** inflammatory bowel disease, ulcerative colitis, integrin αvβ6 antibody, differential diagnosis, disease activity monitoring

## Abstract

**Background/Objectives**: Differential diagnosis between Crohn’s disease (CD) and ulcerative colitis (UC) can be sometimes difficult resulting in the diagnosis of unspecified inflammatory bowel diseases (IBD-U). Data suggest that IgG antibodies against integrin αvβ6 (V6 Ab) help to identify UC patients. Recent studies suggest that measuring V6 Ab serum levels may be valuable for differential diagnostic purposes. The primary objective of the study was to assess the sensitivity and specificity of V6 Ab serum-level measurement in our IBD population to differentiate between colonic/ileocolonic CD and UC with an established diagnosis. Furthermore, we assessed the correlation between disease characteristics, activity and V6 Ab serum levels in UC patients. **Methods**: Consecutive IBD patients with an established diagnosis undergoing control colonoscopy in a tertiary IBD center were included. Baseline demographic data, current treatment, disease extent, clinical, biomarker, endoscopic and histologic disease activity were collected. V6 Ab serum levels were measured with the Anti-Integrin αvβ6 ELISA Kit (RUO). Patients’ written informed consent was obtained. **Results**: A total of 40 IBD patients, including 10 CD and 30 UC patients (15 with clinical activity and 15 in clinical remission) were enrolled. V6 Ab serum levels were significantly higher in UC patients compared to CD (*p* = 0.039). ROC analysis found 1.33 U/mL to be the best cut-off level (*p* = 0.04; AUC: 0.71) with 100% sensitivity and 50% specificity and a positive predictive value of 85.7% and a negative predictive value of 100% to differentiate between UC and CD. No significant correlation was found between V6 Ab serum levels and CRP (*p* = 0.057), fecal calprotectin (*p* = 0.77), endoscopic activity (*p* = 0.624) or disease extent (*p* = 0.624) in UC patients. **Conclusions**: Our study supports the value of V6 Ab serum level measurement as a differential diagnostic tool in IBD patients; however, the optimal cut-off value is yet to be determined. Our data do not support its role in disease activity monitoring.

## 1. Introduction

Inflammatory bowel disease (IBD), including Crohn’s disease (CD) and ulcerative colitis (UC), is a group of chronic, idiopathic inflammatory disorders of the gastrointestinal tract requiring lifelong comprehensive multidisciplinary treatment. The incidence and prevalence of IBD are rising worldwide, with substantial impacts on patient quality of life, healthcare utilization, and long-term morbidity. Chronic inflammation in IBD may lead to complications such as strictures, fistulae, colorectal cancer, and extraintestinal manifestations, necessitating early and accurate diagnosis to guide appropriate therapy.

Diagnosis of CD and UC is based on clinical symptoms, laboratory, imaging, endoscopic, and histological findings. However, clinicians often face difficulties differentiating colonic CD and UC in patients with nonspecific endoscopic and histological features, such as extensive colonic disease with patchy colitis, rectal sparing, atypical ulceration patterns, or mild ileal involvement, resulting in the diagnosis of unspecified IBD (IBD-U). Recent research has focused on the development of additional diagnostic tools to facilitate correct diagnoses.

Optimal biomarkers of IBD are readily measurable and can be objectively and repeatedly assessed in tissues or body fluids such as serum or stool, and are ideally of high sensitivity and specificity to differentiate between UC and CD, as well as cheap and easy to obtain.

ASCA and p-ANCA are the best studied serological markers for differential diagnosis of IBD; however, their sensitivity is relatively low. Serological p-ANCAs can be detected in 6–38% of CD and in 41–73% of UC patients, resulting in low sensitivity for diagnosing IBD (17.2%) and distinguishing UC from CD (52%) [1]. ASCA has been shown to be produced in 59.7% of CD patients, while it is produced in only 13.2% of UC patients, in 10.8% of non-IBD controls, and in 3.2% of healthy controls [2], having moderate sensitivity (37–72%) in identifying patients with Crohn’s disease [1]. Numerous serological markers have since been discovered and investigated for IBD diagnostics or disease progression, such as different anti-glycan antibodies (gASCA, ACCA, ALCA, and AMCA), anti-glycoprotein 2, anti-OmpC, anti-CBir1, and anti-I2. Combinations of these biomarkers have been found to provide higher specificity with lower sensitivity in diagnosing IBD [3,4].

In 2008 Papp et al. presented a panel of a new set of antibodies directed against antiglycans (gASCA, ALCA, ACCA, and AMCA) and a more universal antibody against outer membrane porins (anti-OmpC) aiming to facilitate IBD differential diagnosis and disease phenotype prediction and explore the association of antibody production and NOD2/CARD15 status in an Eastern European cohort of 652 IBD patients. They found the measurement of gASCA and the combination of gASCA and p-ANCA to be the most accurate for differentiating between CD and UC, with 91% specificity and 51–52% sensitivity (AUC 0.761). Moreover, increasing amounts and levels of antibody responses toward gASCA, ALCA, ACCA, AMCA, and OmpC were associated with more complicated disease behavior (*p* < 0.0001) and need for surgery in CD (*p* = 0.023), and a serological dosage effect was also observed. Additionally, gASCA and AMCA antibodies were associated with NOD2/CARD15 with a gene-dosage effect [4]. Furthermore, in 2015, a new prediction model including clinical and serologic markers was demonstrated by the same study group for assessing the probability of developing advanced disease in a prospective cohort of 271 CD patients with non-complicated disease behavior (B1) at diagnosis and a median follow-up of 108 months. The combination of ASCA positivity, ileal disease location, and need for early azathioprin use was associated with the probability of developing advanced disease (need for surgery and disease behavior change) (pLogRank < 0.001) in a 3-, 5-, and 7-year prediction matrix [5].

Despite their suboptimal sensitivity and specificity for establishing a definitive initial diagnosis of IBD, the mentioned serological biomarkers retain utility in discriminating between CD and UC. Their principal clinical relevance, however, lies in their prognostic potential to identify CD patients at increased risk for a complicated disease trajectory, including complicated phenotypes, long-term disease progression, and surgical intervention [3,4,5].

Dysregulation of innate immune signaling, including Toll-like receptor (TLR)-mediated activation and downstream TNF α production, is increasingly recognized as a central driver of mucosal inflammation in IBD. The recent literature also highlights that specific gene expression changes, such as those involving N4BP3, can influence these signaling pathways and contribute to disease pathogenesis by modulating inflammatory cascades in the colonic epithelium [6]. These mechanistic insights support ongoing exploration of epithelial surface molecules, including integrins, as potential targets in UC.

Integrins are transmembrane glycoprotein receptors consisting of α- and β-subunits. They play essential roles in cellular signaling, proliferation, adhesion, and migration [7]. Integrin αvβ6 is expressed on the colonic epithelial cell surface in individuals with ulcerative colitis [8]. It interacts with the extracellular matrix [9] and is reported to play a critical role in preserving epithelial barrier integrity and suppressing epithelial inflammation [10].

In 2021, Kuwada et al. analyzed serum samples of 112 UC patients and 165 healthy controls and found that patients with UC produce IgG antibodies against αvβ6 integrin. They reported a sensitivity of 92.0% and a specificity of 94.8% for the diagnosis of UC. Moreover, anti-integrin αvβ6 antibody (V6 Ab) titers correlated with clinical activity in UC patients [8]. In 2023 Marafini et al. confirmed that serum levels of V6 Ab were significantly elevated in UC patients compared to healthy subjects and CD patients. V6 Ab serum levels were also significantly higher in UC patients compared to CD patients with a colonic-only location [11].

Despite growing interest, the applicability and accuracy of serum V6Ab measurement for differential diagnosis and disease activity monitoring in clinical practice remain to be fully clarified.

## 2. Aims

The primary objective of this cross-sectional study was to assess the sensitivity and specificity of V6 Ab serum-level measurement in discriminating UC vs. colonic/ileocolonic CD patients with an established diagnosis in a tertiary IBD center. Furthermore, we assessed the correlation of clinical, biochemical and endoscopic disease activity and disease characteristics with V6 Ab serum levels.

## 3. Methods

This cross-sectional study included 40 consecutive patients with established IBD diagnosis (10 CD and 30 UC), who underwent a routine control colonoscopy at a tertiary IBD center. The study population was selected to reflect a real-world clinical setting, including both patients in clinical remission and with active disease. Of the 30 UC patients, 15 were in clinical remission and 15 had clinically active disease, as assessed by the partial Mayo score (pMayo). Clinical remission was defined as pMayo ≤ 2, whereas clinical relapse included patients with pMayo ≥ 3. CD patients were identified as negative controls. Disease behavior in CD was characterized according to the Montreal classification. Inclusion criteria for CD patients were colonic or ileocolonic disease location to focus on the differential diagnosis with UC. Baseline data, including demographic characteristics, disease duration, disease extent, current therapy, clinical, biomarker, endoscopic, and histologic disease activity, were collected from electronic medical records. The study design adhered to the treat-to-target approach, wherein symptomatic patients underwent expedited colonoscopy, whereas clinically inactive patients received follow-up colonoscopy every 2–3 years for disease monitoring. Endoscopic activity was assessed using the endoscopic Mayo score (eMayo) in UC and Simple Endoscopic Score for Crohn’s Disease (SES-CD) in CD. Endoscopic remission was defined as eMayo ≤ 1 in UC and SES-CD ≤ 2 in CD. Whole venous blood was obtained at outpatient visits closest to the colonoscopy date. Serum was separated by centrifugation, and aliquots were stored frozen. Anti-integrin αvβ6 antibodies (V6 Ab) were measured using the Anti-Integrin αvβ6 ELISA Kit (Research Use Only) of MBL Ltd. (Fukushima, Japan), following the manufacturer’s instructions. One ELISA Kit enables the analysis of serum samples of 40 patients, and all assays were performed in duplicate to ensure accuracy. Optical density readings were converted to U/mL using a standard curve. Laboratory personnel were blinded to clinical and endoscopic data. The assay’s analytical range, intra- and inter-assay variability, and the method for handling samples below the detection limit were documented to ensure reproducibility. The study was conducted in accordance with the Declaration of Helsinki and approved by the Institutional Scientific Research Ethics Committee of Semmelweis University (SE-RKEB) (206/2023, Date of approval: 6 November 2023).

## 4. Statistics

The continuous variables of the study show a non-Gaussian distribution according to the results of the Shapiro–Wilk normality test. For descriptive purposes, data are characterized as medians and interquartile ranges. For categorical variables, numbers and percentages were used. For comparison of continuous variables, non-parametric tests were used, such as the Mann–Whitney U test or Kruskal–Wallis test with Dunn’s post hoc test. Spearman’s rank correlation tests were performed to determine r values and significance levels. To determine the cut-off value for the anti-integrin level, receiver operating characteristic (ROC) curve analysis was performed, and the area under the ROC curve (AUC) was calculated.

For the statistical analysis and data presentation, IBM SPSS Statistics Version 27.0 (Armonk, NY, USA) and Graph Pad Prism Version 8.0 (San Diego, CA, USA) software were used. *p*-values were calculated, and the significance level was calculated at *p* < 0.05 unless otherwise stated.

## 5. Results

### 5.1. Clinical Characteristics and Disease Activity

A total of 40 IBD patients were enrolled, 10 CD patients with colonic or ileocolonic disease and 30 UC patients, of which 15 were in clinical remission and 15 in clinical relapse. Median age at inclusion (sample collection) was 39 years (range 19–65). In total, 55% (N = 22/18) of patients were males. Median disease duration was 13.5 (IQR 8.75–17.5) years.

Disease location in CD was colonic (L2) in three and ileocolonic (L3) in seven patients, while disease behavior was luminal (B1) in six and stricturing (B2) in four patients. In UC the disease extent was proctitis (E1), left-sided colitis (E2), and pancolitis (E3) in 16.7% (N = 5), 53.3% (N = 16), and 30% (N = 9) of patients according to the Montreal classification. In total,35% (N = 14) of patients were currently taking 5-ASA, 20% (8) corticosteroids, 20% (N = 8) immunosuppressants, and 67.5% (N = 27) biologicals (37.5% (N = 15) anti-TNF, 30% (N = 12) other biological therapy) (Table 1).

Median CDAI in CD patients was 74 (IQR 51–144), while median pMayo in UC patients was 1 in the clinical remission and 4 in the clinically active group (7/15 UC patients showed moderate-to-severe clinical activity). Median CRP level [mg/L] in CD was 4.8 (range 4–51), 4 in clinically inactive UC (range 4–17), and 4 in clinically active UC (range 4–122). FCAL levels were available in 39/40 patients. Median FCAL level [ug/g] was 556 (IQR 239–800) in CD, 40 (IQR 20–693) in clinically inactive UC, and 1000 (IQR 497–1000) in clinically active UC patients. Endoscopy was performed in all 40 patients. Median SES-CD was 8 (range 0–21) in CD; median eMayo was 1 (range 0–2) in clinically inactive UC and 2 (range 1–3) in clinically active UC patients. In UC, 2/15 patients had endoscopically active disease in the clinical remission group and 13/15 in the clinical relapse group.

### 5.2. Serum V6Ab Levels and Their Association with Disease Type, Phenotype, and Activity

**Figure 1:**
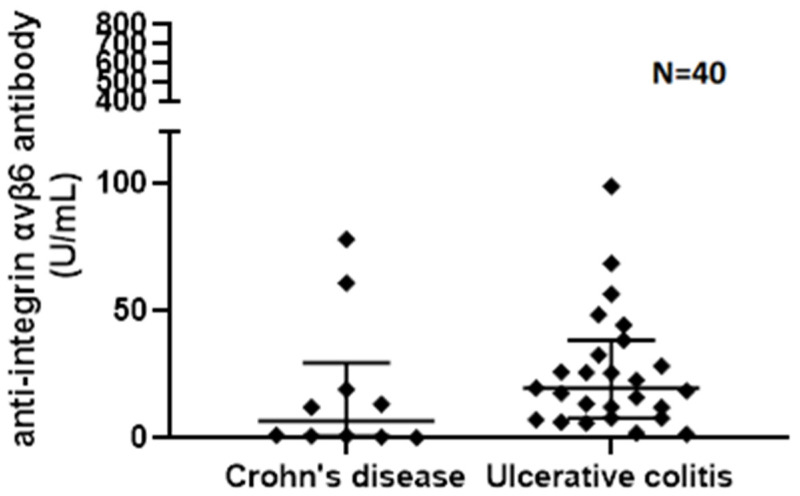
Anti-integrin αvβ6 antibody serum levels measured in Crohn’s disease and ulcerative colitis.

Median V6 Ab serum level [U/mL] was 6.63 (IQR 0.77–29.5) in CD and 21.18 (IQR 8.99–42.91) in UC, 22.74 (IQR 7.17–38.40) in clinical remission, and 19.62 (IQR 7.97–56.62) in clinically active disease. Disease activity and V6Ab levels in the three groups are shown in Table 2. V6 Ab serum levels were significantly higher in UC compared to CD (*p* = 0.04) (Figure 1). However, there was no significant difference in V6 Ab serum levels when comparing CD, clinically active, and inactive UC patients as three separate groups (*p* = 0.117) or in UC between patients in clinical remission or active UC patients (*p* = 0.65). Also, there was no significant difference in V6 Ab serum levels when comparing CD and endoscopically active (eMayo > 1) and inactive (eMayo ≤ 1) UC patients as three separate groups (*p* = 0.108) or endoscopically active and inactive UC patients as two separate groups (*p*= 0.624).

**Figure 2:**
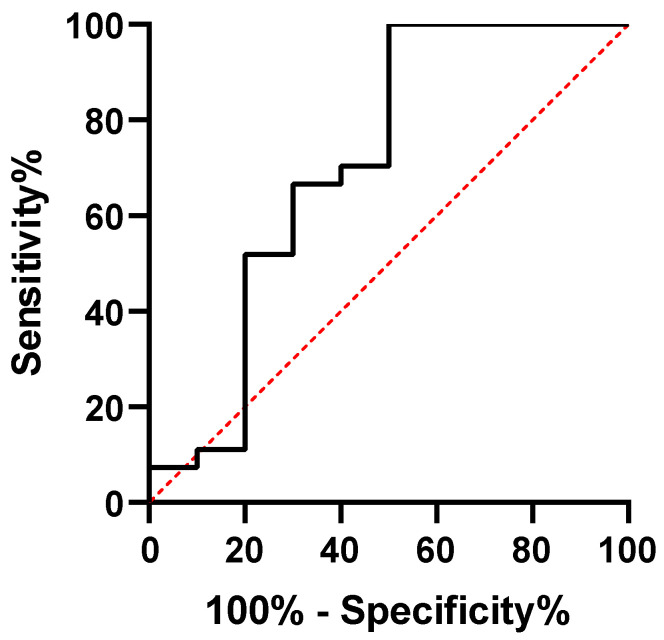
ROC curve of anti-integrin αvβ6 antibody serum levels measurement for diagnosing ulcerative colitis.

ROC analysis found 1.33 U/mL to be the best cut-off level (*p* = 0.0432; AUC: 0.7074 CI 95% 0.4984–0.9373) with 100% (95% CI 87.9–100.0%) sensitivity and 50% (CI 95% 23.7–76.3%) specificity, which translates into a positive predictive value of 85.7% and a negative predictive value of 100% (Figure 2).

**Figure 3:**
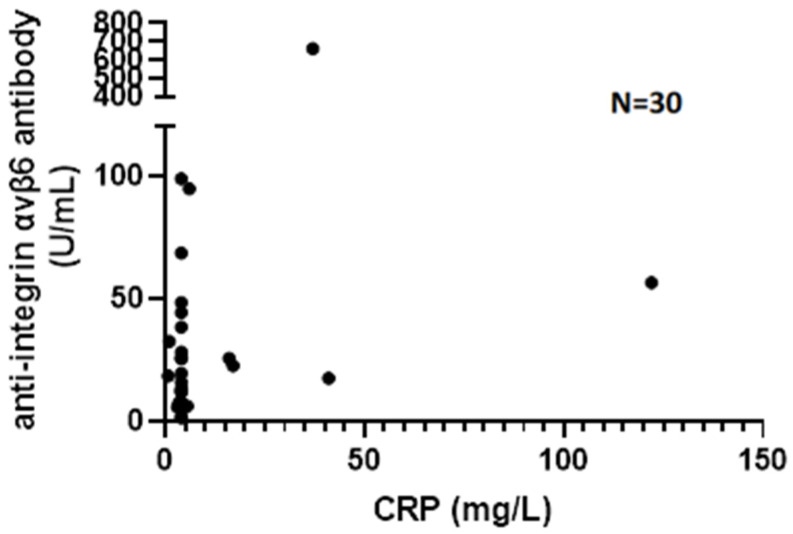
Association of anti-integrin αvβ6 antibody and C-reactive protein serum levels in patients with ulcerative colitis (CRP = C-reactive protein).

**Figure 4:**
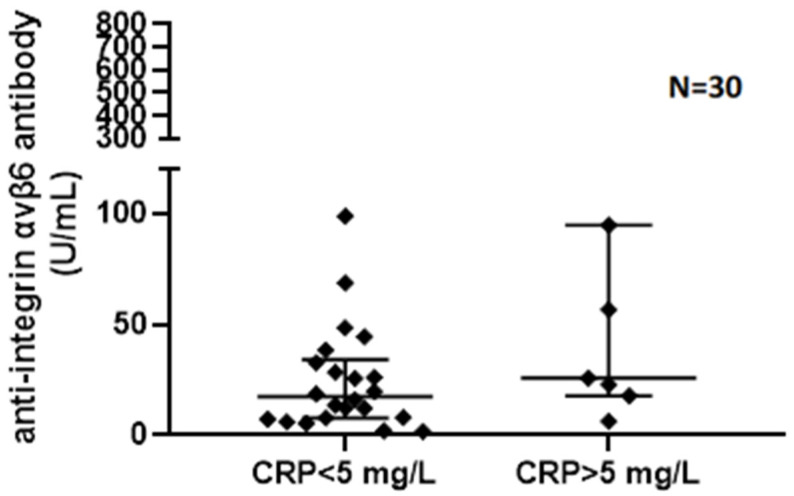
Anti-integrin αvβ6 antibody serum levels measured in ulcerative colitis in patients with normal and elevated C-reactive protein (CRP = C-reactive protein).

**Figure 5:**
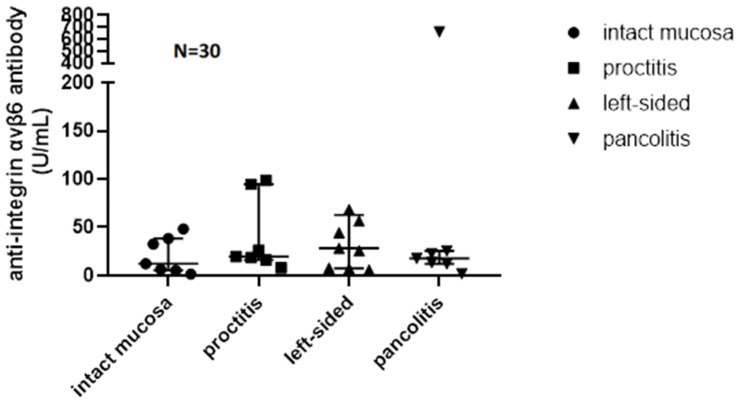
Anti-integrin αvβ6 antibody serum levels measured in ulcerative colitis in patients with different disease extents.

Assessing the correlation of V6 Ab levels with biochemical disease activity in UC patients, we found a weak correlation with CRP (*p* = 0.057, r = 0.35) (Figure 3) and no correlation with FCAL levels (*p* = 0.77, r = −0.08). Comparing biochemically and endoscopically active and inactive UC patients, there was no significant difference in V6 Ab serum levels (FCAL > 250 μg/g, *p* = 0.429; eMayo > 1, *p* = 0.624). Although V6 Ab serum levels were numerically higher in the case of CRP > 5 mg/L (*p* = 0.07) (Figure 4). V6 Ab serum levels showed no correlation with disease extent (normal mucosa/proctitis/left-sided colitis/pancolitis) on endoscopic assessment (*p* = 0.624) (Figure 5).

## 6. Discussion

Differential diagnosis of IBD is challenging in a proportion of IBD patients, especially in cases of extensive severe colitis on endoscopy. The reported prevalence of IBD-U in all IBD patients at diagnosis varies between 3.6% and 9%. Of note, a considerable proportion (app. 25–55%) of patients initially classified as IBD-U are reclassified as UC or CD over time [12,13,14,15]. Such diagnostic uncertainty may delay initiation of targeted therapy and impact long-term outcomes. In addition, colectomy or other surgical interventions performed with an incorrect diagnosis may significantly impact the subsequent disease course and patients’ quality of life. Thus, earlier appropriate diagnosis could contribute to more favorable therapeutic decisions and improve disease outcomes.

Although there have been previous attempts [1,2,3,4,5] to develop serological tests for differential diagnosis of IBD, their insufficient specificity or sensitivity has prevented them from becoming widely adopted, and they have not resolved the existing challenges in differential diagnosis. The most mentioned study from over two decades ago by Joossens et al. investigated the value of serological markers for differential diagnosis in a long-term prospective cohort study of 97 indeterminate colitis patients. Their findings suggested that ASCA+/pANCA− results predict CD in 80% and ASCA−/pANCA+ results predict UC in 63.6% of indeterminate colitis patients [16]. However, the results should be interpreted in the context of the study’s limitations. Most importantly, clinicians responsible for establishing subsequent CD or UC diagnosis were not blinded to the serological results, which may have impacted the clinicians’ evaluation. They also worked with a relatively low case number, considering that only 32% (n = 31) of the followed patients reached a definitive diagnosis during the study period. In addition, 48.5% (n = 47) of the study population tested negative for both biomarkers, and only 48% of seropositive patients were diagnosed with UC or CD over time, resulting in low sensitivity and specificity [16].

V6 Ab serum-level measurement is a promising new tool to distinguish IBD subtypes. The limited data available on V6 Ab serum-level measurement with variable sample sizes suggests reasonable specificity and sensitivity in differentiating between UC and (colonic) CD and other gastrointestinal diseases [8,11,17,18,19,20]. Recently, a nationwide multicenter study was published in Japan to validate the diagnostic value of V6 Ab. A total of 2243 patients with UC, CD, and other gastrointestinal diseases were included. The diagnostic sensitivity of V6 Ab for UC was 87.7%, and its specificities were 82.0% for CD and 87.4% for other gastrointestinal diseases [21].

However, there is no universally accepted value for the optimal cut-off level yet. Moreover, some of the studies presented their results of V6 Ab serum levels only in optical density, whereas statistical methods also differ in the literature. Findings of previous studies are summarized in Table 3.

The cut-off level of 1.33 U/mL calculated in the present study is similar to the cut-off level of 1.64 U/mL defined in the recent nationwide validation study by Okabe et al. [21] and to the 1.68 U/mL found by Bez et al. [19] for differentiating UC and non-UC patients in an observational multicentric study of 228 patients. Notably, Bez et al. [19] performed a separate analysis to determine the optimal cut-off value for differentiating UC from CD patients, which was identified as 1.12 U/mL—an even closer match to our result. Despite the similar cut-off results, we have found a specificity of 50%, which is relatively low compared to the 82.0% [21], 94.4%, and 76% [19] reported in their results. Lower specificity may be explained by the low sample size of the present study. Of note, the inclusion criteria for CD in the present study were patients with colonic or ileocolonic disease location, and in earlier studies [21], colonic CD location was associated with lower differential diagnostic accuracy, which may explain the lower specificity and AUC values found in the present study and Bez et al.’s [19] subanalysis. Furthermore, in the present study, four of the five CD patients with V6 Ab levels above the cut-off, had histological findings of active IBD not specific to UC or CD, and one CD patient was in histological remission.

To maximize specificity for UC over CD, Bez et al. [19] suggested a higher threshold of 13.00 U/mL to best distinguish UC from CD, with a specificity of 90% and sensitivity of 70%. Of note, most studies have excluded IBD-U patients from analyses; thus, the specificity of V6 Ab-level measurement in this population is not available.

The use of V6 Ab serum-level measurement for disease activity monitoring in UC has also been proposed [8,17]. However, the data are inconsistent. Similarly to our results, some studies found no or weak-to-moderate correlation between V6 Ab titers and clinical, biomarker, or endoscopic disease activity [11,19]. Marafini et al. found a significantly higher median level of V6 Ab in patients with pMayo ≥ 2, but no such difference was observed in patients with various disease extents or in the case of FCAL > 250 mg/kg. Also no significant difference was found in patients with V6 Ab levels over or under the defined cut-off in terms of clinical disease activity, fecal calprotectin values, and disease extent [11]. Bez et al. reported weak negative correlation of V6 Ab levels with disease duration (R = −0.22, *p* = 0.03), but not with pMayo (R = 0.12, *p* = 0.24), as well as a weak correlation with FCAL (R = 0.28, *p* = 0.04) and moderate correlation with eMayo (R = 0.60, *p* = 0.03) [19].

In contrast, a proportion of studies reported promising results. Yamamoto et al. conducted a retrospective single-center study with 64 UC patients assessing the association of V6 Ab titers with disease activity. They reported erythrocyte sedimentation rate (ESR) and pMayo, but not CRP, to be associated with V6 Ab titers, also significantly higher titers in patients with eMayo 3 compared to patients in endoscopic remission. A cut-off value of 1.90 U/mL to identify eMayo 0 patients had positive and negative predictive values of 33.3% and 96.3% [22].

Although in Okabe et al.’s [21] validation study, multivariable regression analysis showed that false-positive results in patients with CD were associated with colonic CD, the association of V6 Ab titers with disease extent in UC is not clear. In line with some earlier studies [11,19], we did not find a difference in V6 Ab titers in UC patients with various disease extents (*p* = 0.56), while some studies reported an association with extensive (E3) disease [18].

The use of V6 Ab serum-level measurement for predicting adverse outcomes has also been reported. A significant association between V6 Ab titers and UC-related adverse outcome/disease severity—as a composite of need for biologic therapy, disease extension, systemic steroid use, IBD-related hospitalization, and/or surgery—was reported in a few studies [18,19,22]. Yamamoto et al. found V6 Ab titer to be an independent predictor of treatment escalation in UC [22]. Furthermore, V6 Ab serum levels have been evaluated in UC patients after ileal pouch-anal anastomosis (IPAA) in two Japanese studies [23,24]. According to their results, V6 Ab serum levels decrease in UC patients after IPAA, but they are still higher compared to non-UC controls. In addition, the incidence of pouchitis was significantly higher in patients with high levels of V6 Ab after IPAA, suggesting its role as an indicator of pouchitis.

Of note, production of V6 Ab may precede the clinical diagnosis of UC. In 2023 Livanos et al. measured V6 Ab levels in four longitudinal serum samples collected from 82 subjects who later developed UC and 82 matched controls. V6 Ab titers were significantly higher among individuals who developed UC compared to controls up to 10 years before diagnosis. V6 Ab seropositivity was 2.7% in controls at the four time points, whereas in subjects who later developed UC, it was 12.2% 10 years before diagnosis and increased to 52.4% at the time of diagnosis [18]. Their findings have been confirmed by Sawahashi et al. in 2025 [25].

The strengths of the present study were consecutive patient inclusion and simultaneous clinical, biochemical, and endoscopic activity assessment in all patients. The main limitation of the study is the low patient number, especially that of colonic-only CD patients or CD patients with different endoscopic activities. Furthermore, we investigated patients with established diagnoses of CD and UC; therefore, our data cannot be directly extrapolated to the initial diagnostic setting, particularly with regard to potential application in the IBD-U patient population. Investigating the applicability in IBD-U would require a prospective, long-term follow-up study with a large patient number, allowing evaluation of the test’s accuracy after a definitive UC or CD diagnosis is established. Similar study settings as presented by Joossens et al. [16] in their paper on ASCA and p-ANCA serological markers, with larger patient cohorts and stricter methodologies, would provide much valuable data on applicability in this particular patient population. Additionally, IBD-U does not represent a homogeneous patient group, making it challenging to form a study cohort. It is also possible that in IBD-U patients, differences in pathomechanisms could result in antibody production that differs from that observed in the classical UC or CD forms. Antibody production may also be altered by medication use, especially steroids, immunosuppressants, or biological therapy, which may be a potential confounder that we were not able to address due to the low patient number.

## 7. Conclusions

We conducted a prospective, consecutive, cross-sectional study on 40 IBD patients assessing the value of V6 Ab serum-level measurement for differential diagnosis between CD and UC and disease activity monitoring in UC patients.

Our results confirm the possible role of V6 Ab serum-level measurement as a differential diagnostic tool in IBD patients. V6 Ab serum levels were significantly different in UC and CD patients. However, we found relatively low specificity, with high sensitivity values for UC diagnosis.

In contrast to earlier studies, our data do not support the use of V6 Ab serum-level measurement for disease activity monitoring in UC. Similarly, no significant association was found with biochemical or endoscopic disease activity or disease extent.

Future studies should evaluate V6 Ab in IBD-U populations and its potential integration into multiparametric panels for earlier, more accurate diagnosis and prediction of adverse outcomes or subsequent disease development in IBD patients.

## Figures and Tables

**Table 1:** Baseline characteristics. (UC = ulcerative colitis, CD = Crohn’s disease, IQR = interquartile range, 5-ASA = 5-aminosalicylic acid).

	Total Study Population
Male gender, n (%)	22 (55)
Age at inclusion (years), median [range]	39 [19–65]
Disease duration (years), median [IQR]	13.5 [8.75–17.5]
Treatment, n (%)	
5-ASA	14 (35)
steroids	8 (20)
azathioprine	8 (20)
biologicals	27 (67.5)
anti-TNF	15 (37.5)
vedolizumab	3 (7.5)
other	9 (22.5)
Montreal classification	
Age at onset, n (%) A1/A2/A3	9 (22.5)/25 (62.5)/6 (15)
Disease extension in UC, n (%) E1/E2/E3	5 (16.7)/16 (53.3)/9 (30)
Disease location in CD, n (%) L1/L2/L3	0 (0)/3 (30)/7 (70)
Disease behavior in CD, n (%) B1/B2/B3	6 (60)/4 (40)/0 (0)

**Table 2:** Current disease activity and anti-integrin αvβ6 antibody serum levels in study groups. (UC = ulcerative colitis, CD = Crohn’s disease, pMayo = partial Mayo score, CDAI = Crohn’s disease activity index, IQR = interquartile range, CRP = C-reactive protein, FCAL = fecal calprotectin, SES-CD = Simple Endoscopic Score for Crohn’s Disease, eMayo = endoscopic Mayo score, V6 Ab = anti-integrin αvβ6 antibody).

	Active UC Group	Inactive UC Group	CD Group
Clinical activity			
pMayo, median [IQR]	4 [3–7]	1 [0–1]	-
CDAI, median [IQR]	-	-	74 [51–144]
Biomarker activity			
CRP, median [IQR] (mg/L)	4 [4–5.5]	4 [4–4.75]	4.8 [4–13]
FCAL, median [IQR] (ug/g)	1000 [497–1000]	40 [20–693]	556 [239–801]
Endoscopic activity			
SES-CD, median [range]	-	-	8.5 [0–21]
eMayo, n (%) 0/1/2/3	0 (0)/2 (13)/6 (40)/7 (47)	7 (47)/6 (40)/2 (13)/0 (0)	-
current extension, n (%) E1/E2/E3	7 (47)/2 (13)/3 (20)/3 (20)	0 (0)/5 (33)/6 (40)/4 (27)	-
V6 Ab serum level			
median [IQR] (U/mL)	19.62 [12.00–50.52]	22.74 [9.61–35.50]	6.63 [0.89–17.24]
over cut-off, n (%)	15 (100)	15 (100)	5 (50)

**Table 3:** Summary of studies reporting evidence on sensitivity, specificity, and cut-off values of anti-integrin αvβ6 antibody serum-level measurement. (AUC = area under the curve, HC = healthy controls, N = number of patients, OGD = other gastrointestinal diseases, UC = ulcerative colitis, CD = Crohn’s disease, SD = standard deviation, non-IBD = not Inflammatory Bowel Disease) [8,11,17,18,19,20,21].

Study	N	Cut-Off Level	Sensitivity for UC	Specificity for CD	Specificity for OGD or HC
Okabe [21]	2243	1.64 U/mL	87.7%	82.0%	87.4%
Pertsinidou [20]	570	400 U_A_/L	79.0%	-	94.0% (AUC 0.92)
	473		73.0%	-	93.0% (AUC 0.90)
Rydell [17]	273	95th percentile of healthy controls	76.3%	79.0%	96.1% (AUC 0.95)
Marafini [11]	273	95th percentile of healthy controls	51.9%	83.5%	93.5%
Kuwada [8]	267	mean control + 3 SD	92.0%	-	94.8%
Bez [19]	228	1.68 U/mL 1.12 U/mL	87.9% 89.0%	- 76.0% (AUC 0.89)	94.4% (AUC 0.93) -
Livanos [18]	104	mean non-IBD + 3 SD	70.2%	-	98.1% (AUC 0.99) -
	55		85.5%	-	

## Data Availability

The data presented in this study are available on request from the corresponding author due to privacy restrictions.

## Abbreviations

The following abbreviations are used in this manuscript:

AUC	area under the curve
CD	Crohn’s disease
eMayo	endoscopic Mayo score
ESR	erythrocyte sedimentation rate
FCAL	fecal calprotectin
IBD	inflammatory bowel disease
IBD-U	unspecified inflammatory bowel disease
IPAA	ileal pouch-anal anastomosis
pMayo	partial Mayo score
SES-CD	Simple Endoscopic Score for Crohn’s disease
UC	ulcerative colitis
V6 Ab	anti-integrin αvβ6 antibody
